# Cold-Induced Browning Dynamically Alters the Expression Profiles of Inflammatory Adipokines with Tissue Specificity in Mice

**DOI:** 10.3390/ijms17050795

**Published:** 2016-05-23

**Authors:** Xiao Luo, Ru Jia, Qiangling Zhang, Bo Sun, Jianqun Yan

**Affiliations:** 1 Department of Physiology and Pathophysiology, School of Basic Medical Sciences, Xi’an Jiaotong University Health Science Center, Xi’an 710061, China; xluo@mail.xjtu.edu.cn (X.L.); jiaru123@stu.xjtu.edu.cn (R.J.); langyan.95@163.com (Q.Z.); sunbo1217@mail.xjtu.edu.cn (B.S.); 2 Key Laboratory of Environment and Genes Related to Diseases, Xi’an Jiaotong University, Ministry of Education of China, Xi’an 710061, China; 3 Department of Prosthodontics, Stomatological Hospital, College of Stomatology, Xi’an Jiaotong University, Xi’an 710061, China

**Keywords:** inflammatory adipokines, beige cells, cold exposure, β_3_-adrenoceptor agonist, depot-specificity

## Abstract

Cold exposure or β_3_-adrenoceptor agonist treatment induces the adipose tissues remodeling, relevant for beige adipogenesis within white adipose tissue (WAT). It remains unclear whether this process influences inflammatory adipokines expression in adipose tissues. We determine the temporal profile of cold or β_3_-adrenoceptor agonist (CL316,243)-induced changes in the expression of inflammatory adipokines in adipose tissues in mice or primary mice adipocytes. Male C57BL/6J mice at eight weeks old were exposed to 4 °C for 1–5 days. Interscapular brown adipose tissue (iBAT), inguinal subcutaneous WAT (sWAT) and epididymal WAT (eWAT) were harvested for gene and protein expression analysis. In addition, cultured primary mice brown adipocyte (BA) and white adipocyte (WA) treated with or without CL316,243 were harvested for gene expression analysis. The inflammatory adipokines expressed significantly higher in WAT than BAT at baseline. They were rapidly changed in iBAT, while down-regulated in sWAT and up-regulated in eWAT during the cold acclimation. Upon CL316,243 treatment, detected inflammatory adipokines except *Leptin* were transiently increased in both BA and WA. Our *in vivo* and *in vitro* data demonstrate that the browning process alters the inflammatory adipokines expression in adipose tissues, which is acutely responded to in iBAT, dynamically decreased in sWAT whilst increased in eWAT for compensation.

## 1. Introduction

Adipose tissue is an essential component of our body, the excessive accumulation of adipose tissue will lead to obesity that predisposes to hypertension, diabetes, cardiovascular diseases and so on. In recent years, it is well established that adipose tissue is not only working as an energy storage depot, but also increasingly viewed as an active endocrine organ with a high metabolic activity secreting numerous adipocyte-derived hormones referred to as adipokines [1,2]. These adipokines play a major role in regulating food intake, energy metabolism and inflammatory response acting on the brain, liver, muscle and other tissues via autocrine, paracrine and endocrine pathways [3,4]. It is thought that the imbalance between the inflammatory adipokines leads to monocyte/macrophage infiltration and a vicious cycle which eventually results in systemic inflammation compromising cardiac, vascular and metabolic tissues [5,6]. Hence, understanding the expression profile and mechanisms controlling of these inflammatory adipokines will provide potential therapeutic opportunities to tackle the global epidemic of metabolic disease.

The adipose tissue in mammals has been divided into two types. The white adipose tissue (WAT) stores excess energy in the form of triglycerides. Conversely, the brown adipose tissue (BAT) is specialized in energy expenditure and responsible for non-shivering thermogenesis called adaptive thermogenesis [7,8]. What is more, recent studies have identified an intermediate type of adipocytes which exist within certain WATs in mice and rats [9]. These cells are named beige or brite (brown in white) cells and they express the high level of uncoupling protein-1 (UCP-1) and mitochondria genes, and also show the multilocular lipid droplets like the morphological characteristic of BAT [10,11]. These cells become more prominent upon prolonged stimulation by cold or β_3_-adrenoceptor agonists such as CL316,243 [12]. Compared to BAT, WAT is the major source of inflammatory adipokines [13,14,15]. One of the most important inflammatory adipokines is the 16 kDa cytokine-like protein, Leptin, which is the product of the obese gene (ob) and has a variety of physiological effects [16]. It helps to keep energy balance through the inhibition of food intake and the consumption of energy expenditure [4,17]. Besides, Leptin also acts on multiple types of immune cells, such as neutrophils, monocytes/macrophages and T cells, to promote the release of inflammatory cytokines [18,19,20,21].

Another important adipokine, Adiponectin (also known as AdipoQ), contributes to enhance insulin sensitivity in obese mouse [22], normalize lipid metabolism dysfunction, inhibit energy expenditure and lead to weight loss in diet-induced insulin-resistance mice [23]. Apart from its metabolic functions, Adiponectin is also implicated in the regulation of immune responses. Many lines of evidence show that Adiponectin has anti-inflammatory functions due to its modulation of macrophage phenotype [14,24]. Additional adipokines include interleukin-10 (Il-10), Monocyte chemoattractant protein–1 (Mcp-1) and Tumor necrosis factor-α (Tnf-α). These adipokines are classified into anti-inflammatory and pro-inflammatory depending on their biological properties.

Since the previous studies of our group have indicated that cold-induced remodeling of WAT acquired some characteristics of BAT, including the thermogenic capacity and molecular morphology [25], we propose that the expression profile of inflammatory adipokines in WAT will also be “browning” and get some features of BAT during the beige adipogenesis. Hence, the main objective of this study is to investigate the expression levels of main inflammatory adipokines in both BAT and WAT during cold exposure. The effect of browning by β_3_-adrenoceptor agonist (CL316,243) on the expression of main inflammatory adipokines has also been assessed in primary mice brown adipocytes (BA) and white adipocytes (WA) *in vitro*.

## 2. Results

### 2.1. Inflammatory Adipokines Expression Levels Exhibit Depot-Specificity in Mouse Adipose Tissues

**Figure 1:**
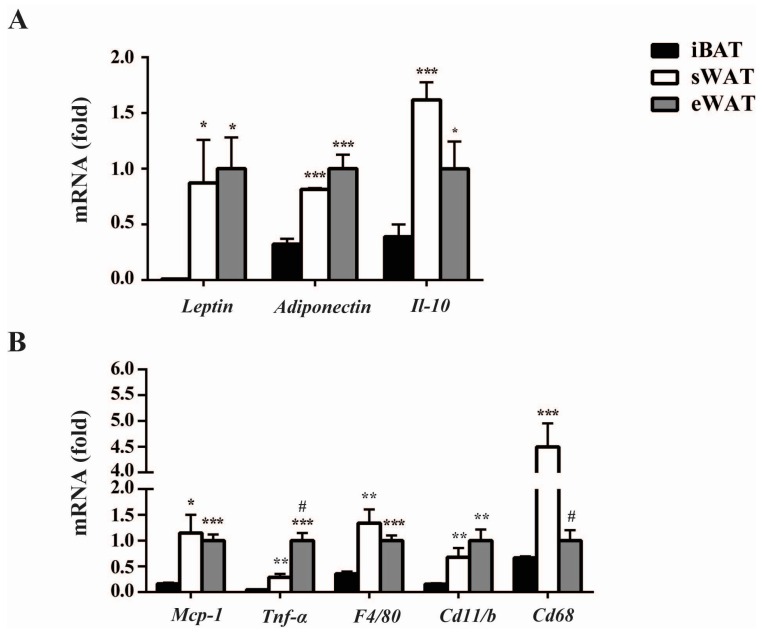
Inflammatory adipokines expression levels exhibit depot-specificity in mice adipose tissues at baseline (in room temperature without any treatment). Quantitative PCR analysis of anti-inflammatory (**A**) and pro-inflammatory (**B**) adipokines genes expression in iBAT, sWAT and eWAT at baseline. The data show the fold changes of the expressions for the target genes in interscapular brown adipose tissue (iBAT), inguinal subcutaneous WAT (sWAT) and epididymal WAT (eWAT) of RT (room temperature) mice (*n* = 6 for each group). Values are mean ± S.E.M. and expression of genes is corrected for the housekeeping gene *Cyclophilin*. (* *p* < 0.05, ** *p* < 0.01, *** *p* < 0.001 *vs.* the expression level of iBAT, while # *p* < 0.05 *vs.* the expression level of sWAT.)

To determine the expression levels of important inflammatory adipokines in the different adipose tissues under the normal physiological level, we detected the mRNA levels of those in interscapular brown adipose tissue (iBAT), inguinal subcutaneous WAT (sWAT) and epididymal WAT (eWAT) of mice under room temperature using RT-qPCR analysis. We found that the expression levels of these adipokines were depot-specific among the different adipose tissues. The mRNA levels of *Leptin*, *Adiponectin* and *Il-10* in sWAT and eWAT were significantly higher than in iBAT. Especially for *Leptin*, the mRNA level in sWAT and eWAT was 10-fold higher than in iBAT. Moreover, these adipokines expressions between sWAT and eWAT showed no statistically significant difference (Figure 1A). In addition, the expression levels of pro-inflammatory adipokines, including *Mcp-1*, *Tnf-α*, and the macrophages markers, *F4/80*, *Cd11b* and *Cd68*, were all much higher in both sWAT and eWAT than in iBAT (Figure 1B). Interestingly, *Tnf-α* expression was significantly higher in eWAT than in sWAT, but this condition reversed in the expression of *Cd68.* These results indicated that the abundant adipokines expressed differently at baseline of adipose tissues, with the significantly higher levels in WAT than those in iBAT universally.

### 2.2. Cold Exposure Differentially Alters the Expressions of Inflammatory Adipokines in Adipose Tissues

**Figure 2:**
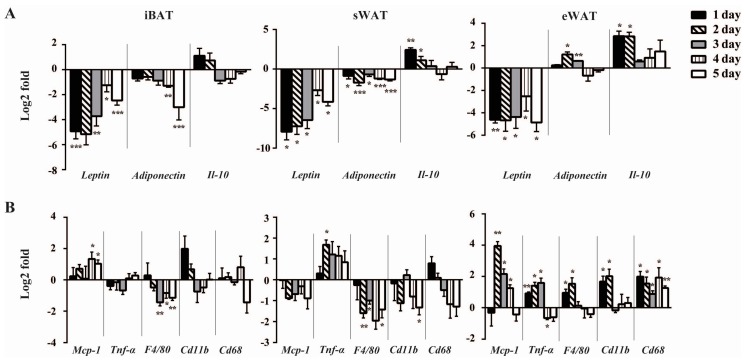
Cold exposure differentially alters the expression of inflammatory adipokines among adipose tissues. Quantitative PCR analysis of anti-inflammatory (**A**) and pro-inflammatory (**B**) adipokines genes expression in iBAT, sWAT and eWAT of control mice and mice exposed to Cold (4 °C) up to 5 day. The data show the fold changes of the expression for the target genes in iBAT, sWAT and eWAT of RT and Cold mice (*n* = 6 for each group). Values are mean ± S.E.M. and expression of genes is corrected for the housekeeping gene *Cyclophilin*. All the data were normalized to the expression in RT mice respectively to show the cold-induced gene expression differences. Erected bars: genes up-regulated and inverted bars: genes down-regulated compared to the RT mice. (* *p* < 0.05, ** *p* < 0.01, *** *p* < 0.001).

Given that inflammatory adipokines exhibited depot-specific expression pattern and cold induced browning of adipose tissues [25,26,27], we investigated whether the expression profiles of inflammatory adipokines in adipose tissues also showed the browning-like transformation. The expression of *Leptin* exhibited a rapid and sustained down-regulation across all the three depots since the first day of the cold exposure. *Adiponectin*, expressed highly and specifically in mature adipocytes, showed interesting and remarkable changes during the cold acclimation. In iBAT, it tended to decrease gradually from the first day, but only reached the statistical significance on Days 4–5. In sWAT, *Adiponectin* was decreased significantly throughout the study. While unlike that in iBAT and sWAT, *Adiponectin* in eWAT was increased at Days 2–3 of the cold treatment, but came back to the baseline on subsequent days. Interestingly, *Il-10* showed the similar expression trend in sWAT and eWAT, which was up-regulated at Days 1–2 but decreased to basal level on following days, however, in iBAT, there was no significant change in *Il-10* expression throughout the study (Figure 2A). Whilst *Leptin*, *Adiponectin* and *Il-10* expression levels reflect inflammatory status of adipose tissues [28,29] we did not observe any change in macrophage infiltration in adipose tissues (data not shown).

Having detailed the temporal depot-specific profiles of anti-inflammatory adipokines following cold exposure, we extended our investigation to examine pro-inflammatory adipokines (Figure 2B). *Mcp-1*, which is involved in macrophages infiltration, was up-regulated in iBAT from Day 4 of cold exposure. Despite failing to reach statistical significance, the *Mcp-1* expression tended to decrease in sWAT following cold exposure. In eWAT, *Mcp-1* expression was increased by around four-fold on Day 2, then decreased slightly on Days 3–4 but remained significantly higher than that of the RT group. Interestingly, *Tnf-α* level in sWAT was increased transiently only at Day 2 of the cold exposure. In eWAT, it was up-regulated persistently during Days 1–3 and decreased dramatically at Day 4, whilst no significant change was observed in iBAT. Different from *Tnf-α*, *F4/80* was down-regulated in iBAT and sWAT from Day 3 and 2, respectively. However, in eWAT, it was up-regulated on the first two days of cold stimulation and then decreased to basal level thereafter. Similarly, cold exposure did not change the expression of *Cd11b* in iBATh, whilst it induced a brief decrease at Day 5 in sWAT. In contrast, eWAT *Cd11b* levels were increased sustainably. We also found that the expressions of *Cd68* were unchanged in both iBAT and sWAT during cold exposure, but rapidly increased by around two-fold at Days 1–2 in eWAT. These data demonstrated that the temporal profiles of these inflammatory markers during cold exposure differed across the three depots.

Overall, the expression levels of inflammatory adipokines in sWAT were decreased mostly during the cold exposure. It is somewhat unexpected that the expression profile of inflammatory adipokines was increased universally in eWAT and more complex in iBAT with the time of cold. To summarize, these cold-induced changes in gene expression profiles of inflammatory adipokines demonstrated that the remodeling of adipose tissues in response to cold result in a depot-specific alteration of the inflammatory adipokines expression at different time points.

### 2.3. Cold Exposure Changes Adiponectin and Leptin Protein Levels in Adipose Tissues

**Figure 3:**
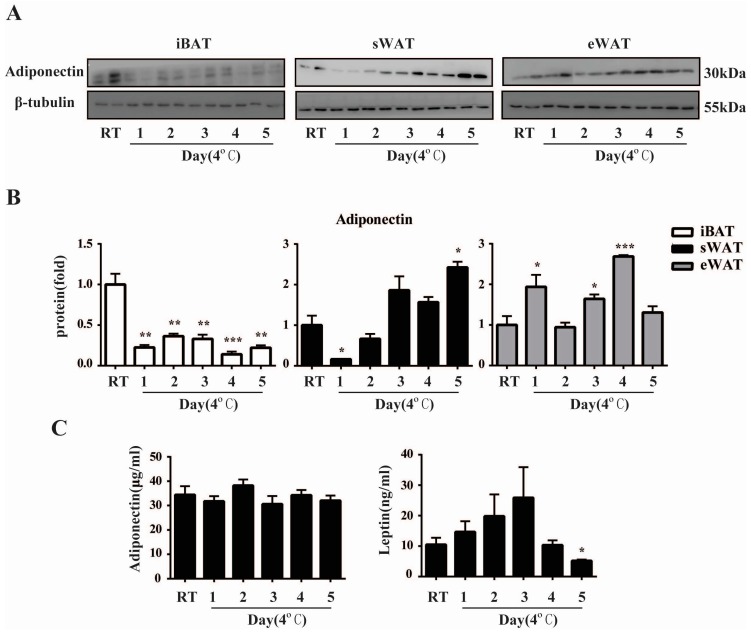
Cold exposure changes Adiponectin and Leptin protein levels in adipose tissues in mice. (**A**) Western blot analysis for Adiponectin using total protein isolated from iBAT, sWAT and eWAT of RT and Cold mice. (*n* = 6 for each group); (**B**) Quantification of Western blot analysis. Protein content is expressed relative to the control and represents three independent experiments with triplicate observations in each experiment. Volume is the sum of all pixel intensities within a band. All data are normalized to β-tubulin and are expressed as mean ± S.E.M. (* *p* < 0.05, ** *p* < 0.01, *** *p* < 0.001); (**C**) Plasma Adiponectin and Leptin levels in RT and Cold mice were determined by ELISA. (*n* = 6 per group) All data are presented as mean ± S.E.M. * *p* < 0.05, ** *p* < 0.01 for the Cold compared to the RT group.

To extend our observations at the genetic level, we characterized the temporal effects of cold exposure on the expressions of Adiponectin protein in different depots using Western blot and detected its plasma concentration using ELISA. Consistent with the gene expression in iBAT, Western blot analysis demonstrated that Adiponectin protein was decreased significantly in iBAT throughout the cold exposure (Figure 3A,B). In sWAT, the expression of Adiponectin protein dropped at Day 1 and then increased gradually to a significant higher level at Day 5 of the cold exposure. Similar to its gene expression, Adiponectin protein was also up-regulated during the cold acclimation in eWAT. Perhaps surprisingly, the alterations of plasma Adiponectin levels did not reach statistical significance compared to the RT mice throughout the study. Besides, unlike the rapid and sustained decrease of *Leptin* gene expression during the cold exposure, plasma Leptin levels did not decline significantly until Day 5 compared to the control group, indicating that the changes in circulating level of Leptin lagged far behind that in the localized adipose tissues (Figure 3C).

### 2.4. CL316,243 Treatment Alters the Expression of Inflammatory Adipokines in Brown Adipocyte (BA) and White Adipocyte (WA)

**Figure 4:**
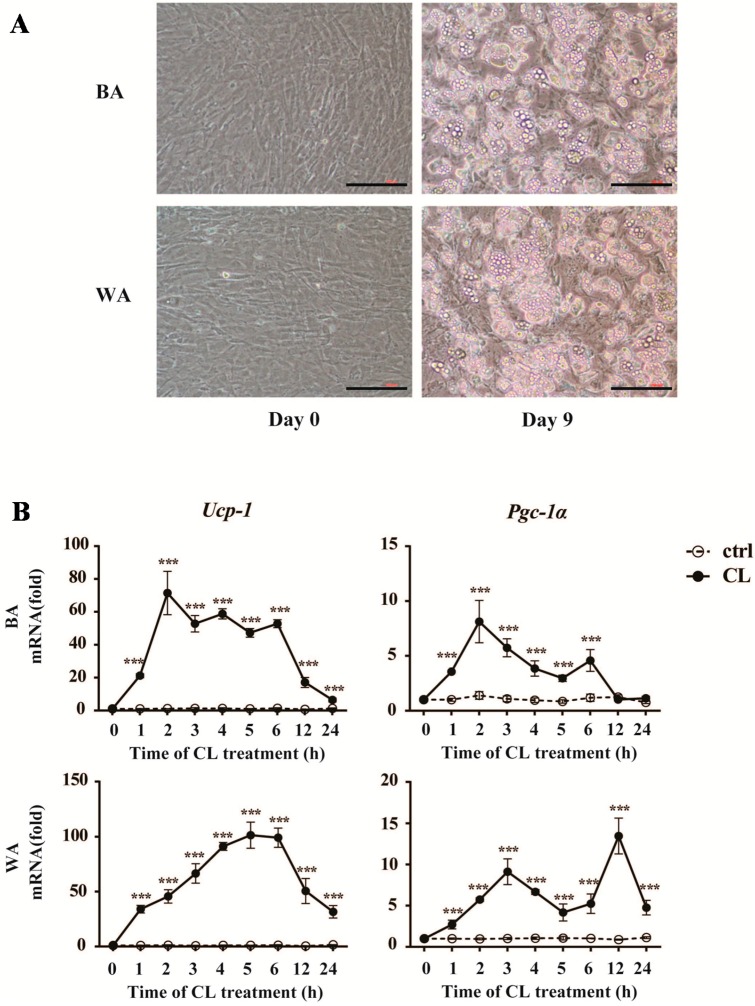
Browning transition with increase of browning markers in the cultured mature Brown Adipocyte (BA) and White Adipocyte (WA) after CL316,243 treatment. (**A**) Morphology of the brown and white adipocytes before (Day 0) and after (Day 9) the differentiation. The mature adipocytes after nine days of differentiation were used for CL316,243 treatment. Scale bar: 200 µm; (**B**) Quantitative PCR analysis of Ucp-1 and Pgc-1α gene expression in cultured mice brown and white adipocytes after CL316,243 treatment up to 24 h. The data show the fold changes of the expression for the target genes in BA and WA at Hour 0. Values are mean ± S.E.M. from three independent experiments. The expression of genes is corrected for the housekeeping gene *Cyclophilin* (*** *p* < 0.001).

To further verify the effects of early cold exposure on the changes of inflammatory adipokines in different adipose tissues, we conducted the experiment on cultured adipocytes to observe the expression profiles of inflammatory adipokines during the browning process within 24 h *in vitro*. In the present study, we provided clear evidences for the differentiated status of adipocytes in our *in vitro* study and the browning process in cultured adipocytes as shown in Figure 4A,B. Most of the preadipocytes were differentiated enough for the induction of browning at Day 9. The expression of *Ucp-1*, the core thermogenic gene, was induced rapidly by CL316,243 within one hour and kept several tens of times higher than that of the control group at all time points in both BA and WA. The expression of α subunit of peroxisome proliferators-activated receptor-γ coactivator 1 (*Pgc-1α*), a key inducer of BA activation, had the same tendency as *Ucp-1* in WA. However, in BA, it was up-regulated during the first six hours upon CL316,243 treatment, and came back to the baseline after 12 h.

**Figure 5:**
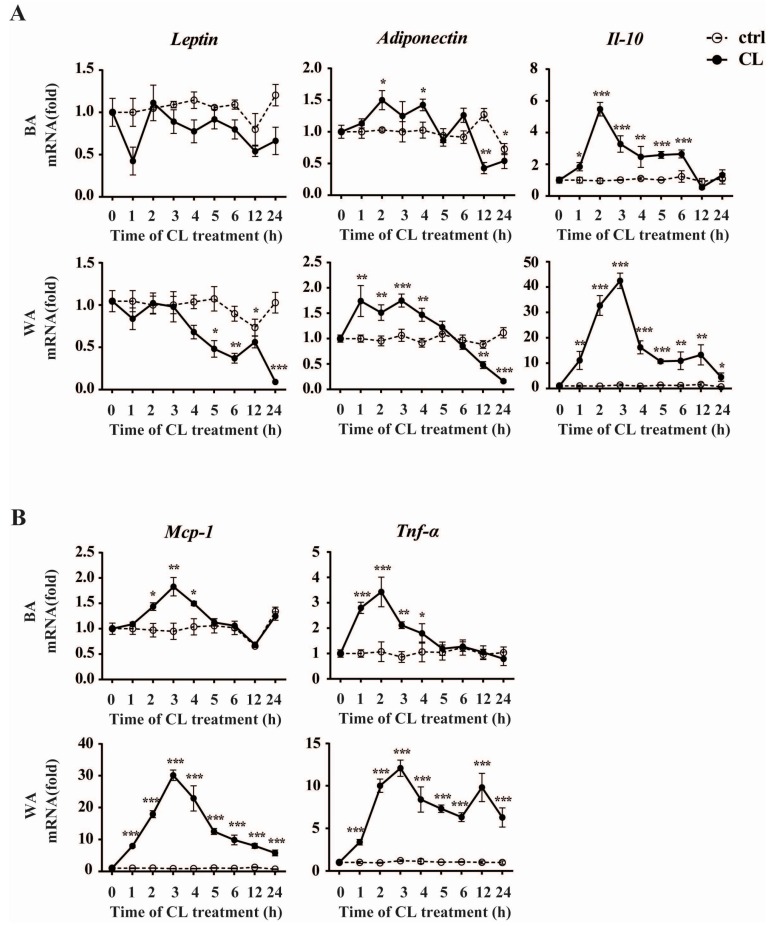
CL316,243 treatment alters the expressions of inflammatory adipokines in BA and WA. Quantitative PCR analysis of anti-inflammatory (**A**) and pro-inflammatory (**B**) adipokines expression in cultured mice BA and WA after CL316,243 treatment up to 24 h. The data show the fold changes of the expression for the target genes in BA and WA at Hour 0. Values are mean ± S.E.M. from three independent experiments. The expression of genes is corrected for the housekeeping gene *Cyclophilin* (* *p* < 0.05, ** *p* < 0.01, *** *p* < 0.001).

We then investigated the expressions of key inflammatory adipokines within the 24-h treatment of CL316,243 (Figure 5). Interestingly, the expression of *Adiponectin* underwent an obvious reversal both in BA and WA, which was increased within first few hours and then declined significantly after 12 h of the treatment. We have also found that, different from expressed *in vivo*, the alteration of *Leptin* in BA did not reach statistical significance during the whole browning process. While in WA, *Leptin* decreased significantly from Hour 5 onwards. Similar to the expression pattern of *Pgc-1α*, *Il-10* was always up-regulated during the treatment in WA. While in BA, this increased level only maintained the first half of the experimental process. These results were consistent with that *in vivo*, indicating the very early effects of *Il-10* during the browning process. Furthermore, we also detected the two key pro-inflammatory adipokines *Mcp-1* and *Tnf-α*, and found their expression profiles were similar to each other. As shown in Figure 5B, both of the two markers increased first and did not fall to the baseline untilHour 5 in BA. While in WA, *Mcp-1* and *Tnf-α* expression levels were strongly induced across all time points during the experiment. These expression profiles in white and brown adipocytes in response to CL316,243 further reinforce the notion that the inflammatory adipokines expression during the browning process exhibits depot-specificity and temporal differences.

## 3. Discussion

It is well known that cold exposure or β_3_-adrenoceptor agonist would induce the browning of adipose tissues with the beige adipogenesis. However, the origin of beige cells within WAT and gene expression pattern was distinct from those of BAT [26,27]. Recently, we reported that the time course effects of the cold-induced browning on adipose tissues exhibited depot-specificity [25]. It remains poorly documented whether the expression profiles of inflammatory adipokines also alter differently during the browning process. In this study, we have addressed this by characterizing the temporal changes in expression of inflammatory adipokines induced by 1–5 days of cold exposure in mouse iBAT, sWAT and eWAT. In addition, the expressions of those in BA and WA in response to β_3_-adrenoceptor agonist (CL316,243) have also been studied. We find that WAT exhibits a dominant source of inflammatory adipokines and plays a central role in the regulation of systemic metabolism under basal condition. While during the browning process, the expressions of inflammatory adipokines were dynamically changed both *in vivo* and *in vitro*, with depot-specificity among the adipose tissues.

As the biggest endocrine organ in the body, adipose tissues product and secrete various adipokines [15,30]. Different kind of adipose tissue depots can be distinguished by their profile of secretion of adipokines [14]. The current notion in the field is that WAT is the main source of inflammatory adipokines [31]. Our studies support this view when the expression levels of inflammatory adipokines in sWAT and eWAT are compared with that of iBAT.

Leptin, primarily secreted by adipose tissues, plays an important role in regulating energy balance and body mass [32]. Our present data suggested that *Leptin* mRNA levels decreased significantly since the early stage of browning process, which was consistent with the previous studies [33,34,35,36]. However, we observed the decreased plasma level of Leptin only at Day 5. This result was consistent with the previous study that the Leptin in serum did not alter upon the acute cold exposure [37] but significantly decreased after the chronic cold acclimatization [38]. Collectively, our data indicated the hysteresis quality of the alteration in the systemic level of Leptin.

Similar expression pattern was observed in Adiponectin. We have shown that the expression profile of *Adiponectin* mRNA was decreased significantly in sWAT and iBAT, but up-regulated in eWAT. However, Adiponectin protein was increased in sWAT at Day 5 of cold exposure. This discordance between mRNA and protein levels of Adiponectin was possibly due to the low efficiency of protein biosynthesis in the cold environment in sWAT. Interestingly, in the early phase of browning process, there was a reversal of *Adiponectin* gene expression in both white and brown adipocytes, which was up-regulated first and then declined significantly after 12 h treatment of CL316,243 *in vitro*. There is also another report which claimed that chronic cold exposure induced Adiponectin expression in subcutaneous fat [39]. The reasons for the discordant observations are more likely to be due to the different control groups of mice (30 °C *versus* RT, which is below thermoneutrality for mice). However, it is noteworthy that the plasma levels of Adiponectin did not fluctuate during cold exposure. This was in line with the previous study which demonstrated a significant decrease in *Adiponectin* mRNA in adipose tissues after cold exposure or β_3_-agonists treatment but no alteration in serum Adiponectin level [40,41]. These observations might be attributed to the following reasons. First, adipose tissue is both the primary site of Adiponectin synthesis and a major target organ for Adiponectin actions so that the main regulatory manner of Adiponectin is autocrine or paracrine, but not endocrine. Second, the expression of Adiponectin on plasma level might also exhibit hysteresis quality after the gene level. Taken together, these findings highlight a complicated role of Adiponectin response to cold or β_3_-agonists, which needs to be further investigated in the future.

In addition to our recent report that there were regional differences in the fat mass, cellular morphology and browning markers among the adipose tissues in response to cold [25], the present study showed the alteration of inflammatory adipokines expression during the browning was depot-specific either. Although the expression of *Il-10* and *Tnf-α* was unchanged in iBAT during the cold exposure, their expression in BA *in vitro* was enhanced and then came down to the basal line within 24 h, indicating that the expression of some inflammatory adipokines was activated rapidly at the very early stage of the browning process. However, the inflammatory adipokines in sWAT showed different expression profile. Except for *Tnf-α*, whose expression exhibited a rising trend both *in vivo* and *in vitro*, the expression level of inflammatory adipokines (especially the pro-inflammatory adipokines) was up-regulated within the first few hours and then decreased in the following days in sWAT, which showed a similar expression profile of inflammatory adipokines in iBAT. This might be attributed to the important role of sWAT as an endocrine organ which participates in the long-range general regulation of metabolism. It is tempting to speculate that the modulation of the endocrine function in sWAT was in favor of the inflammatory balance during the browning process. Interestingly, the expression of many inflammatory adipokines differs between sWAT and eWAT upon cold exposure. In spite of the down regulation of inflammatory adipokines in sWAT, the expressions of such adipokines in eWAT were obviously increased during the cold stimulation. This finding is not surprising given the well-documented different propensity to accumulate beige cells between sWAT and eWAT and the differences in adipocyte biology in rodents [25,42]. It is also consistent with the thesis that WAT is regionally distinct in terms of function, adipokines production, and inflammation; even the density of solitary adipose tissue macrophages in sWAT is much lower than that in eWAT [43]. Moreover, the decrease expression of inflammatory adipokines in sWAT may induce the compensatory increase of that in eWAT. As an important part of visceral fat, eWAT is thought to play an important role in the etiology of various metabolic disorders [44,45,46,47,48]. Understanding the mechanisms of WAT/BAT phenotypic conversion in eWAT might point the way toward novel therapies for these diseases. Therefore, the role of eWAT in the modulation of inflammation under cold exposure still warrants further investigation.

All these present observations essentially accord with the findings that cold exposure helps to prevent obesity, insulin resistance and other metabolic disorders. It is based on the regulation in endocrine and metabolism during the cold-induced beige adipogenesis among the adipose tissues [26,39,49]. While someone found that people living in northern hemisphere are fatter because of cold climate. We think this contradictory phenomenon may be due to the perennial rather than the acute cold acclimation, which still needs additional study.

## 4. Materials and Methods

### 4.1. Mouse Colonies and Cold Exposure

All male mice were in the C57BL/6J background, and obtained from the Medical Experimental Animal Center of Xi’an Jiaotong University (Xi’an, China) at 8 weeks of age. They were singly kept in a specific pathogen-free (SPF) environment with a 12:12-h light–dark cycle and had free access to water and standard chow diet. After one week of adaption, they were randomly divided into six groups: room temperature (RT) for 5 days, and cold exposure (Cold) for 1–5 days (*n* = 6 for each group), as in our previous study [25]. All animal studies were approved by the Ethical Committee of Xi’an Jiaotong University, China. This study was approved by the Institutional Animal Care and Use Committee of Xi’an Jiaotong University (Number: XJTU-2012-03-06-0037). Upon completion of the experiment, the blood of mice was collected by retro-orbital bleed. The mice were subsequently sacrificed and the iBAT, sWAT and eWAT [42,50] were removed and frozen in liquid nitrogen for RNA and protein extraction.

### 4.2. Adipocyte Culture

For the culture of primary mice BA and WA, iBAT and sWAT were isolated from 3-week-old C57BL/6J male mice. The white preadipocytes were maintained in DMEM/F12 (Gibco–BRL Laboratories, Grand Island, NY, USA) supplemented with 2 mM l-glutamine, 100 U/mL penicillin (Sigma, St. Louis, MO, USA) and 10% fetal bovine serum (FBS) (Gibco–BRL Laboratories), while the brown preadipocytes were maintained in DMEM high glucose medium (Gibco–BRL Laboratories) supplemented with 5 mM HEPES, 100 U/mL penicillin (Sigma) and 20% FBS (Gibco–BRL Laboratories) both at 37 °C and 5% CO_2_. To induce differentiation, postconfluent preadipocytes were cultured in a standard differentiation medium (the DMEM or DMEM/F12 contained 850 nM insulin and 1 nM triiodothyronine). For the first three days of differentiation period, 0.5 mM 3-isobutyl-1-methylxanthine, 0.125 mM indomethacin, 1 µM dexamethasone and 1 µM Rosiglitazone were also added. The cells were fully differentiated after 9 days of culture in the differentiation medium.

The cells were treated with CL316,243 (2 µM, Tocris Bioscience, Bristol, UK) for 1, 2, 3, 4, 5, 6, 12 and 24 h after the deprivation of FBS for 12 h. After CL316,243 treatment, mature adipocytes were lysed in Trizol for RNA extraction.

### 4.3. Quantitative Real-Time PCR

Trizol (Invitrogen, San Diego, CA, USA) was used to isolate the total mRNA of both tissues and cells. The mRNA samples were reverse transcribed into cDNA using a commercial RT-PCR Kit according to the manufacturer’s instructions (Thermo scientific, Waltham, MA, USA). Relative PCR quantification was performed using a commercial RT-PCR Kit according to the manufacturer’s instructions (TaKaRa, Japan). Expression data were normalized to the amount of *Cyclophilin* mRNA using the –ΔΔ*C*_t_ method. Primers synthesized by AUGCT (Beijing, China) are listed in Table 1.

### 4.4. Western Blot Analysis

Previously described procedures were used [51]. Briefly, protein samples (20 µg) were separated using 10% SDS-PAGE gels, then transferred to polyvinylidene difluoride membranes (Millipore, Bedford, MA, USA). Membranes were blocked with 5% nonfat dry milk in TBS containing 0.1% Tween for 1 h at room temperature and then blotted with primary antibodies (anti-Adiponectin (1:500) (#2789) (Cell Signaling Technology, Inc., Beverley, MA, USA), anti-β-tubulin (1:1000) (sc-9104) (Santa Cruz Biotechnology Inc., Santa Cruz, CA, USA) overnight. After washing, membranes were incubated with a secondary horseradish peroxidase (HRP)-coupled antibody and visualized using Immobilon HRP substrate (Millipore). The density of the bands was quantified using ImageJ Software (National Institute of Health, Bethesda, MD, USA). The ratio of the intensity of the target protein to that of β-tubulin loading control was calculated to represent the expression level of the protein.

### 4.5. Plasma Adipokines Measurement

The plasma sample from each mouse was obtained by centrifuging at 900× *g* in 4 °C for 15 min after collection. The extracted plasma was frozen at −80 °C until analysis. Concentrations of Adiponectin and Leptin in plasma samples were measured using the Mouse Adiponectin and Leptin ELISA kit (Thermo scientific) on an automatic imark Microplate Absorbance Reader (Bio-Rad Laboratories, Hercules, CA, USA) according to manufacturer’s procedures.

### 4.6. Statistical Analysis

Statistical analyses were performed with GraphPad Prism 6.0 (GraphPad Software, La Jolla, CA, USA). Values were expressed as means ± S.E.M. of independent experiments. Comparisons between the two groups were analyzed by paired Student’s *t* tests. Comparisons among groups were made by one-way ANOVA test. Differences were considered significant when *p* < 0.05.

## 5. Conclusions

In summary, we show that iBAT is not the dominant source of inflammatory adipokines compared to sWAT and eWAT. Moreover, *in vivo* and *in vitro* studies suggest that the expression of inflammatory adipokines during browning process is very complex and different among adipose tissues, which was acutely responded in iBAT, meanwhile dynamically decreased to the benefit of inflammatory harmony in sWAT, but also significantly increased in eWAT.

## Figures and Tables

**Table 1:** Primers used for quantitative real-time PCR analysis.

Gene	Forward Primer (3′–5′)	Reverse Primer (3′–5′)	Size (bp)
*Ucp-1*	CTGCCAGGACAGTACCCAAG	TCAGCTGTTCAAAGCACACA	148
*Pgc-1α*	CCCTGCCATTGTTAAGACC	TGCTGCTGTTCCTGTTTTC	161
*Leptin*	GGGCTTCACCCCATTCTGA	TGGCTATCTGCAGCACATTTTG	103
*Adiponectin*	GATGGCACTCCTGGAGAGAA	TCTCCAGGCTCTCCTTTCCT	143
*Il-10*	AGCTCCAAGACCAAGGTGTC	TCCAAGGAGTTGTTTCCGTTA	195
*Mcp-1*	AGGTCCCTGTCATGCTTCTG	GCTGCTGGTGATCCTCTTGT	167
*F4/80*	TGGATGAGTGCTCCAGGAAT	GATGGCCAAGGATCTGAAAA	126
*Cd11b*	CGGAAAGTAGTGAGAGAACTGTTTC	TTATAATCCAAGGGATCACCGAATTT	113
*Cd68*	CTTCCCACAGGCAGCACAG	AATGATGAGAGGCAGCAAGAGG	235
*Tnf-α*	ACGTGGAACTGGCAGAAGAG	GGCCATAGAACTGATGAGAGG	200
*Cyclophlin*	CATACAGGTCCTGGCATCTTGTC	AGACCACATGCTTGCCATCCAG	112

## Abbreviations

BA	brown adipocyte
eWAT	epididymal white adipose tissue
iBAT	interscapular brown adipose tissue
Il-10	interleukin-10
Mcp-1	monocyte chemoattractant protein-1
PGC-1α	α subunit of peroxisome proliferators-activated receptor-γ coactivator 1
RT	room temperature
RT-qPCR	quantitative real-time PCR
sWAT	subcutaneous white adipose tissue
Tnf-α	tumor necrosis factor-α
Ucp-1	uncoupling protein-1
WA	white adipocyte
